# Supplementary material for: Structural snapshots of La Crosse virus polymerase reveal the mechanisms underlying Peribunyaviridae replication and transcription
Source: Nat Commun. 2022 Feb 16;13:902. doi: 10.1038/s41467-022-28428-z (PMC8850483; doi:10.1038/s41467-022-28428-z)
Supplement: Supplementary file 1 — Supplementary Information [file 41467_2022_28428_MOESM1_ESM.pdf]

SUPPLEMENTARY INFORMATIONS

Structural snapshots of La Crosse virus polymerase reveal the mechanisms underlying *Peribunyaviridae* replication and transcription

Benoît Arragain *et al.*

Supplementary Table 1. Cryo-EM data collection, refinement and validation statistics of LACV-L replication snapshots

	**LACV L Replication initiation** (PDB 7ORN) (EMD-13043)	**LACV L Replication early elongation** (PDB 7ORO) (EMD-13044)	**LACV L Replication late-elongation** (PDB 7ORI) (EMD-13038)
**Data collection and processing**			
Microscope	Thermo Fisher Scientific Titan Krios	Thermo Fisher Scientific Glacios	Thermo Fisher Scientific Glacios
Camera	Gatan K3	Gatan K2 Summit	Gatan K2 Summit
Magnification	130000	36000	36000
Voltage (kV)	300	200	200
Number of frames	40	60	60
Electron exposure (e^-/A^2)	50	60	60
Defocus range (μm)	-0.8 to -1.8	-0.8 to -2.0	-0.8 to -2.0
Pixel size (Å)	0.645	1.145	1.145
Symmetry imposed	C1	C1	C1
Initial/Final micrographs (no.)	15573/15444	2927/2923	1848/1841
Final particles (no.)	641008	417757	24151
Map resolution (Å) 0.143 FSC threshold	2.8	2.9	3.9
Map resolution range (Å)	2.6-3.5	2.7-4	3.6-6
**Refinement**			
Initial model used	6Z6G	6Z8K	6Z8K
Model resolution (Å) 0.5 FSC threshold	2.8	3.0	3.9
Map sharpening B factor ($Å^2$)	-50	-50	-50
Model composition			
Protein residues	1700	2008	2007
Nucleotide residues	35	48	44
Ligands	3	2	3
Water	78	0	0
B-factor ($Å^2$, min-max (mean))			
Protein	38.17-142.20 (64.15)	37.78-160.62 (74.97)	68.43-192.36 (109.75)
Nucleotides	44.49-108.37 (67.92)	51.26-142.63 (75.16)	97.23-188.85 (112.03)
Ligands	51.03-53.69 (51.85)	46.51-133.86 (90.19)	88.19-172.55 (112.03)
R.m.s deviations			
Bond lengths (Å)	0.003	0.004	0.003
Bond angles (°)	0.508	0.531	0.501
Validation			
MolProbity score	1.37	1.52	1.69
Clashscore	5.27	6.40	9.32
Poor rotamers (%)	0.06	0.05	0.00
Ramachandran plot			
Favored (%)	97.57	97.04	96.79
Allowed (%)	2.43	2.96	3.21
Disallowed (%)	0	0	0

Supplementary Table 2. Cryo-EM data collection, refinement and validation statistics of LACV-L transcription snapshots

	**LACV L Transcription cleavage conformation** (PDB 7ORJ) (EMD-13039)	**LACV L Capped primer active site entry** (PDB 7ORK) (EMD-13040)	**LACV L Transcription initiation** (PDB 7ORL) (EMD-13041)	**LACV L Transcription early elongation** (PDB 7ORM) (EMD-13042)
**Data collection and processing**				
Microscope	Thermo Fisher Scientific Glacios	Thermo Fisher Scientific Titan Krios	Thermo Fisher Scientific Titan Krios	Thermo Fisher Scientific Glacios
Camera	Gatan K2 Summit	Gatan K3	Gatan K3	Gatan K2 Summit
Magnification	36000	130000	130000	36000
Voltage (kV)	200	300	300	200
Number of frames	60	40	40	60
Electron exposure (e^-/A^2)	60	50	50	60
Defocus range (μm)	-0.8 to -2.0	-0.8 to -1.8	-0.8 to -1.8	-0.8 to -2.0
Pixel size (Å)	1.145	0.645	0.645	1.145
Symmetry imposed	C1	C1	C1	C1
Initial/Final micrographs (no.)	3270/3149	15573/15444	15573/15444	2524/2363
Final particles (no.)	29065	76579	19112	229480
Map resolution (Å) 0.143 FSC threshold	3.9	3.1	3.6	3.3
Map resolution range (Å)	3.6-6.0	2.8-5	3.3-5	3.1-5.5
**Refinement**				
Initial model used	6Z6G	6Z6G	6Z6G	6Z8K
Model resolution (Å) 0.5 FSC threshold	3.9	3.0	3.6	3.4
Map sharpening B factor ($Å^2$)	-60	-50	-50	-80
Model composition				
Protein residues	2129	2182	2183	2145
Nucleotide residues	36	37	47	40
Ligands	2	4	3	2
Water	0	48	0	0
B-factor ($Å^2$, min-max (mean))				
Protein	18.86-240.60 (60.25)	12.50-164.40 (60.74)	54.53-210.52 (97.25)	19.07-204.31 (61.26)
Nucleotides	29.50-93.79 (55.70)	34.94-154.19 (66.57)	76.53-172.59 (124.10)	29.40-152.41 (66.03)
Ligands	37.90-99.40 (49.45)	31.21-140.20 (49.15)	63.41-98.72 (97.56)	25.00-112.91 (68.95)
R.m.s deviations				
Bond lengths (Å)	0.003	0.004	0.002	0.003
Bond angles (°)	0.579	0.533	0.560	0.533
Validation				
MolProbity score	1.72	1.59	1.72	1.61
Clashscore	9.89	6.94	6.48	7.63
Poor rotamers (%)	0.00	0.01	0.00	0.00
Ramachandran plot				
Favored (%)	96.73	96.73	96.18	96.85
Allowed (%)	3.27	3.27	3.73	3.10
Disallowed (%)	0	0	0	0

Supplementary Table 3 Primers

Primers for constructs used in LACV minigenome assays		
**Construct**	**Forward**	**Reverse**
pTM-LACV_L_Nter	gaaccacggggacgtggttttcctttg	cccttgcagtattaatcctagccaagaattgttgatactcttgatagtccatagcactagcgtagtcgggcacg
pTM-LACV_L_Cter	cactaaactgatgaaaaggaaggtgggaggagtaatatagagtttgatgctagctacccatacgatgttccagattacg	gctttgttagcagccggatcgtcgac
pTM-LACV_L_Ci doubleStrep	gaggcaattgaagcattagctgcagaaggatatggagggtcttggagtcacccccagttcgag	gctttgccaagtgaaagcttttcaattttttctagattactctcccctccagaagcactagcgtagtcgggcac

Primers for constructs used for expression, purification, in vitro activity assays and electron microscopy		
**Construct**	**Forward**	**Reverse**
LACV-L_{CItag} (CI tag insertion)	agtggatggagccacccacaattcgagaaaggtagcggatacgaaagcaacctgggtaaaatcg	tccgctacctttctcgaattgtgggtggctccatccacttccttccgtagccaaagcctc
LACV-LCItag_H34K (H34K mutation)	gtcgacctgctgatggcccgtaaggactatttcggacgcgaactctg	cagagttcgcgtccgaaatagtccttacgggccatcagcaggtcgac
LACV-$L_{CItag\_H34K\_M989A}$ (M989A mutation)	cgttgcaaactcaacccagacgaagctattagcgagccgggagacggtaa	cttaccgtctcccggctcgctaatagcttcgtctgggttgagtttgcaacg
LACV-LCItag_H34K_I990A (I990A mutation)	gcaaactcaacccagacgaaatggctagcgagccgggagacggtaagctg	ctttcagcttaccgtctcccggctcgctagccatttcgtctgggttgagtttgc
LACV-$L_{CItag\_H34K\_S991A}$ (S991A mutation)	ctcaacccagacgaaatgattgctgagccgggagacggtaagctgaaag	ctttcagcttaccgtctcccggctcagcaatcatttcgtctgggttgag

Primer used for In vitro transcription		
**Construct**	**Forward**	**Reverse**
37-mer	taatacgactcactatagg	tatctatacttggtagtacactactattatagcatcctatagtgagtcgtatta
21-mer	taatacgactcactatagg	uacactactattatagcatcctatagtgagtcgtatta
14-mer	taatacgactcactatagg	actatatagcatcctatagtgagtcgtatta

Supplementary Table 4 Reagents

REAGENT or RESOURCE	SOURCE	IDENTIFIER
Bacterial and Virus Strains		
DH10EMBacY *Escherichia coli* cells	Nie et al., 2014	N/A
Chemicals, Peptides, and Recombinants Proteins		
X-tremeGENE HP DNA Transfection reagent	Sigma-Aldrich	Cat.# 6366244001
NanoFectin Transfection Reagent	GE-Healthcare (PAA)	Cat.# Q050-005
Opti-MEM Reduced serum-free medium	Gibco-BRL	Cat.# 31985-070
Express Five SFM	ThermoFisher	Cat.# 10486025
Sf-900 II SFM	ThermoFisher	Cat.# 10902161
L-Glutamine (200mM)	ThermoFisher	Cat.# 25030149
Bluo-Gal	Sigma-Aldrich	Cat.# B2904
cOmplete EDTA-free Protease Inhibitor	Roche	Cat.# 118361700001
d-Desthiobiotin	Sigma-Aldrich	Cat.# D1411
Adenosine 5′-triphosphate disodium salt hydrate	Sigma-Aldrich	Cat.# A2383
Cytidine 5′-triphosphate disodium salt	Sigma-Aldrich	Cat.# C1506
Uridine 5′-triphosphate trisodium salt hydrate	Sigma-Aldrich	Cat.# U6625
Guanosine 5′-triphosphate sodium salt hydrate	Sigma-Aldrich	Cat.# G8877
GTP, [α-32P]- 3000Ci/mmol 10mCi/ml, 250 µCi	PerkinElmer	Cat.# NEG506H-250UC
Decade markers system	ThermoFisher	Cat.# AM7778
Critical Commercial Assays		
Dual-Luciferase Reporter Assay system	Promega	Cat.# E1910
NucleoSpin Gel and PCR Cleanup	Macherey-Nagel	Cat.# 740609.50
Gibson Assembly Master Mix	NEW ENGLAND BioLabs	Cat.# E2611S
Vaccinia capping system	NEW ENGLAND BioLabs	Cat.# M2080S
RNase T1	ThermoFisher	Cat.# AM2283
Experimental Models: Cells Lines		
HEK-293	ThermoFisher	Cat.# 85120602
Trichoplusia ni High Five Cells in Express Five Medium	ThermoFisher	Cat.# B85502

Spodoptera frugiperda Sf21 cells in Sf-900 II SFM	ThermoFisher	Cat.# 11497013
Others		
StrepTrap HP 5 mL column	Sigma-Aldrich	Cat.# 17-5247-01
HiTrap Heparin HP 5 mL column	Sigma-Aldrich	Cat.# 17-0407-01
Superdex 200 Increase 10/300 GL	Sigma-Aldrich	Cat.# 28-9909-44
Amicon Ultra (10 kDa cut-off)	Merck Millipore	Cat.# UFC501096
UltraAuFoil gold grids 300 mesh, R 1.2/1.3	Quantifoil	N/A

Oligonucleotides	SOURCE	NAME
5’-AGUAGUGUGCUACCAAG-3’	Microsynth	5’ vRNA 1-17
5’-ACGAGUGUCGUACCAAG-3’	Microsynth	5’ 1-17 Bpm
5’-UAUCUAUACUUGGUAGUACACUACU-3’	Microsynth	3’ vRNA 1-25
5’-AACGUUAUCUAUACUUGGUAGUACACUACU-3’	Microsynth	3’ vRNA 1-30
(m7Gppp)GAAUGCUAUAAUAG	TriLink Biotechnologies	Cap 14-mer AG
5’-TAATACGACTCACTATAGG-3’	Eurofins	T7 promoter
5’-TATCTATACTTGGTAGTACACTACTATTATAGCATCCTATAGTGAGTCGTATTA-3’	Eurofins	37-mer for *in vitro* transcription
5’-ACTATATAGCATCCTATAGTGAGTCGTATTA-3’	Eurofins	14-mer AGU for *in vitro* transcription

Supplementary Table 5. Search patterns used to calculate the read numbers in Supplementary Fig. 2

Replication

<table>
<tr><th>Type</th><th>Remarks</th><th>Search Settings</th><th>Search pattern</th></tr>
<tr><td>Reads containing the replication products</td><td>filter for length between 22-32</td><td>-k 4s</td><td>AGTAGTGTACTACCAAGTATAGATA</td></tr>
<tr><td>Replication Products</td><td></td><td>-k 0</td><td>^AGTAGTGTA</td></tr>
<tr><td>GGU/GU 5' extended replication products</td><td></td><td>-k 0</td><td>^(GT|GGT)AGTAGTGTA</td></tr>
<tr><td>Replication products with a G mismatch in 5'</td><td></td><td>-k 0</td><td>^GGTAGTGT</td></tr>
</table>

Transcription

Type	Remarks	Search Settings	Search pattern
Transcript, no realignment		-k 4s	"^GGAATGCTATAATAGTAGTGTACTACCAAGTATAGATA" ; "ATA.{0,6}$"
Transcript, one realignment	three rounds of search and a length filter >40 <48	-k 4s; NA ; -k 0	"^GGAATGCTATAATAGTAGTAGTGTACTACCAAGTATAGATA" ; "ATA.{0,6}$" ; ".+AGTAGTAGTGTA.+"
Transcript, two realignments	three rounds of search and a length filter >40	-k 4s; NA ; -k 0	"^GGAATGCTATAATAGTAGTAGTAGTGTACTACCAAGTATAGATA"; "ATA.{0,6}$" ; ".+AGTAGTAGTAGTGTA.+"
Transcript, three realignments	three rounds of search and a length filter >40	-k 4s; NA ; -k 0	"^GGAATGCTATAATAGTAGTAGTAGTAGTGTACTACCAAGTATAGATA"; "ATA.{0,6}$"; ".+AGTAGTAGTAGTAGTGTA.+"
Rest	length filter >36 <41	NA	NA

SUPPLEMENTARY FIGURE 1

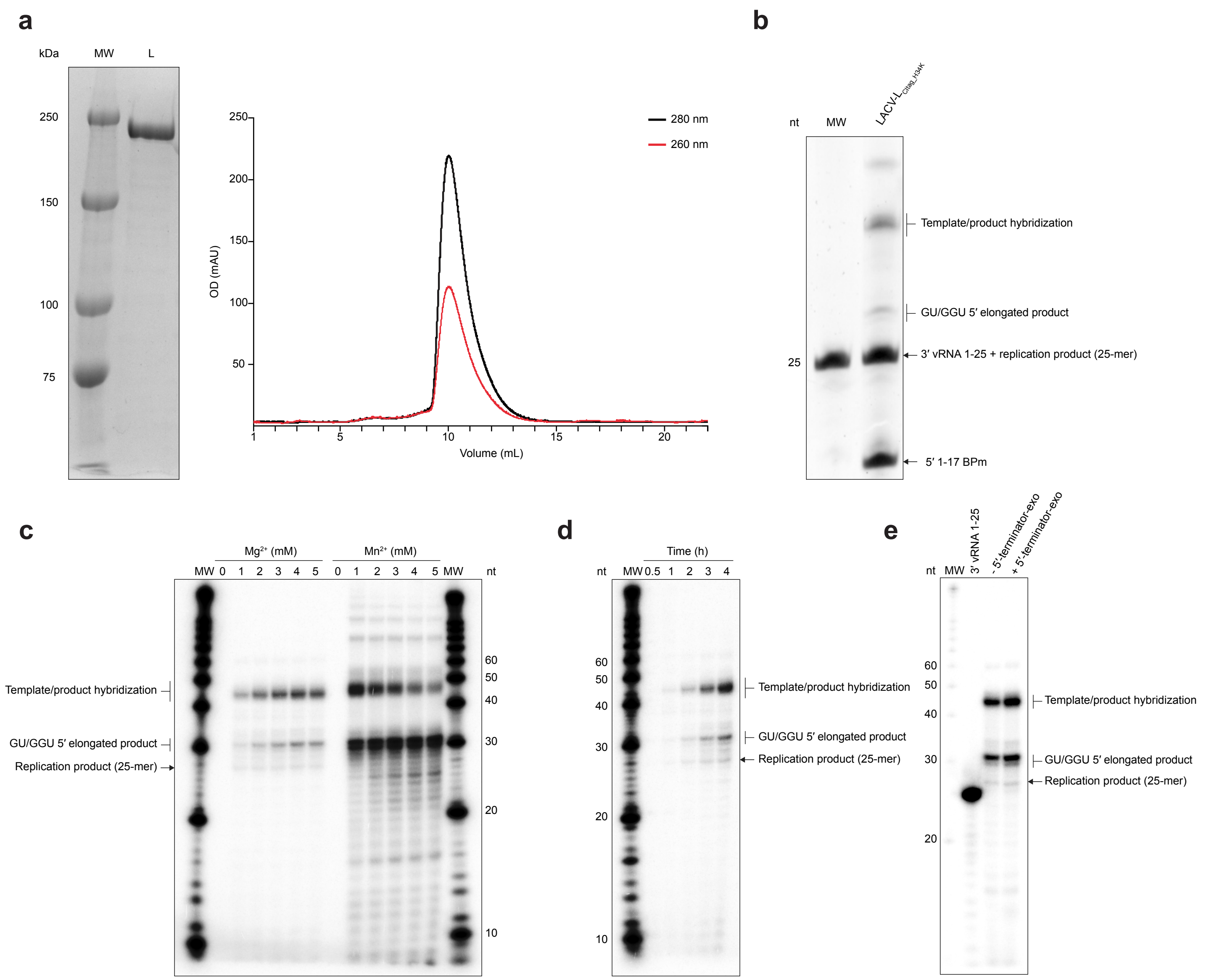

Supplementary Fig. 1. LACV-L$_{\mathrm{CItag_H34K}}$ purification and replication activity optimization

a, 6% SDS-PAGE gel of LACV-L$_{\mathrm{CItag_H34K}}$ (MW: Molecular weight; L: LACV-L$_{\mathrm{CItag\text{-}H34K}}$) and the corresponding gel filtration profile. Absorbance curves at 280 and 260 nm are indicated and respectively colored in black and red. Source data are provided as a Source Data file. This experiment was repeated independently 10 times with similar results.

b, Urea-PAGE gel stained with SYBR-Gold showing a molecular weight marker (MW) of 25 nucleotides next to LACV-LCItag_H34K replication assay done in presence of 3′-vRNA1-25, 5′-1-17BPm and 4 nucleotides. Source data are provided as a Source Data file. This experiment was repeated independently 2 times with similar results.

c, Optimization of the divalent metal ion concentration for LACV-L$_{\mathrm{CItag_H34K}}$ replication activity. Assessment of replication product formation using concentration ranging from 0 to 5 mM of $MgCl_2$ or $MnCl_2$. The molecular weight marker (MW) corresponds to the decade marker. Source data are provided as a Source Data file. This experiment was repeated independently 2 times with similar results.

d, Time course of LACV-L$_{\mathrm{CItag_H34K}}$ replication activity. Reactions were stopped after 0.5, 1, 2, 3 or 4h. The molecular weight marker (MW) corresponds to the decade marker. Source data are provided as a Source Data file. This experiment was repeated independently 2 times with similar results.

e, LACV-L replication reaction products incubated or not with 5′ Terminator exonuclease (5′-terminator-exo) that specifically degrades 5′ mono-phosphate RNA shown on a 20% Urea-PAGE gel. The molecular weight marker (MW) corresponds to the decade marker. Source data are provided as a Source Data file. This experiment was repeated independently 2 times with similar results.

SUPPLEMENTARY FIGURE 2

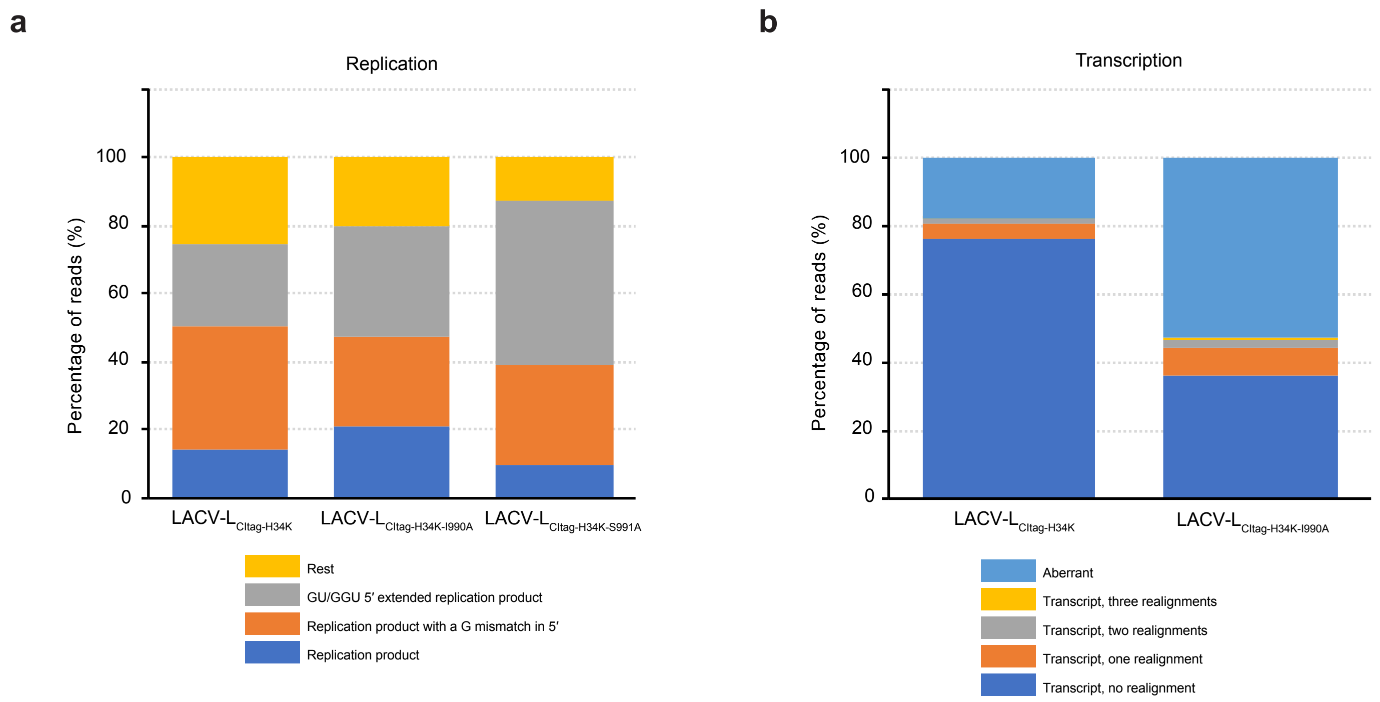

Supplementary Fig. 2. RNA-sequencing

a, NGS analysis of reads containing the replication products for LACV-L$_{CItag-H34K}$, LACV-L$_{CItag-H34K-I990A}$ and LACV-L$_{CItag-H34K-S991A}$. The percentage of read categories is shown. The replication products displayed in blue correspond to the expected 25-mer products. Replication products where the expected 5′ terminal nucleotide is replaced by G are displayed in orange. Replication products that are extended in 5′ by GU or GGU sequences are shown in gray. The other reads that contain the replication products but do not fall in the above categories are shown in green.

b, NGS analysis of reads containing the capped primer with a length compatible with transcription products. The percentage of read categories is shown. The 37-mer transcripts containing the capped primer and the expected product are shown in blue. The realigned transcripts that display one, two and three AGU nucleotide triplet(s) inserted between the primer and the product are shown in orange, gray and yellow, respectively. The aberrant products containing the capped primer and have a length compatible with transcription products (with or without realignment) but do not contain the full product are shown in light blue. Most of these latter reads correspond to the capped primer followed by multiple AGU repeats corresponding to successive realignments that did not lead to product formation.

SUPPLEMENTARY FIGURE 3

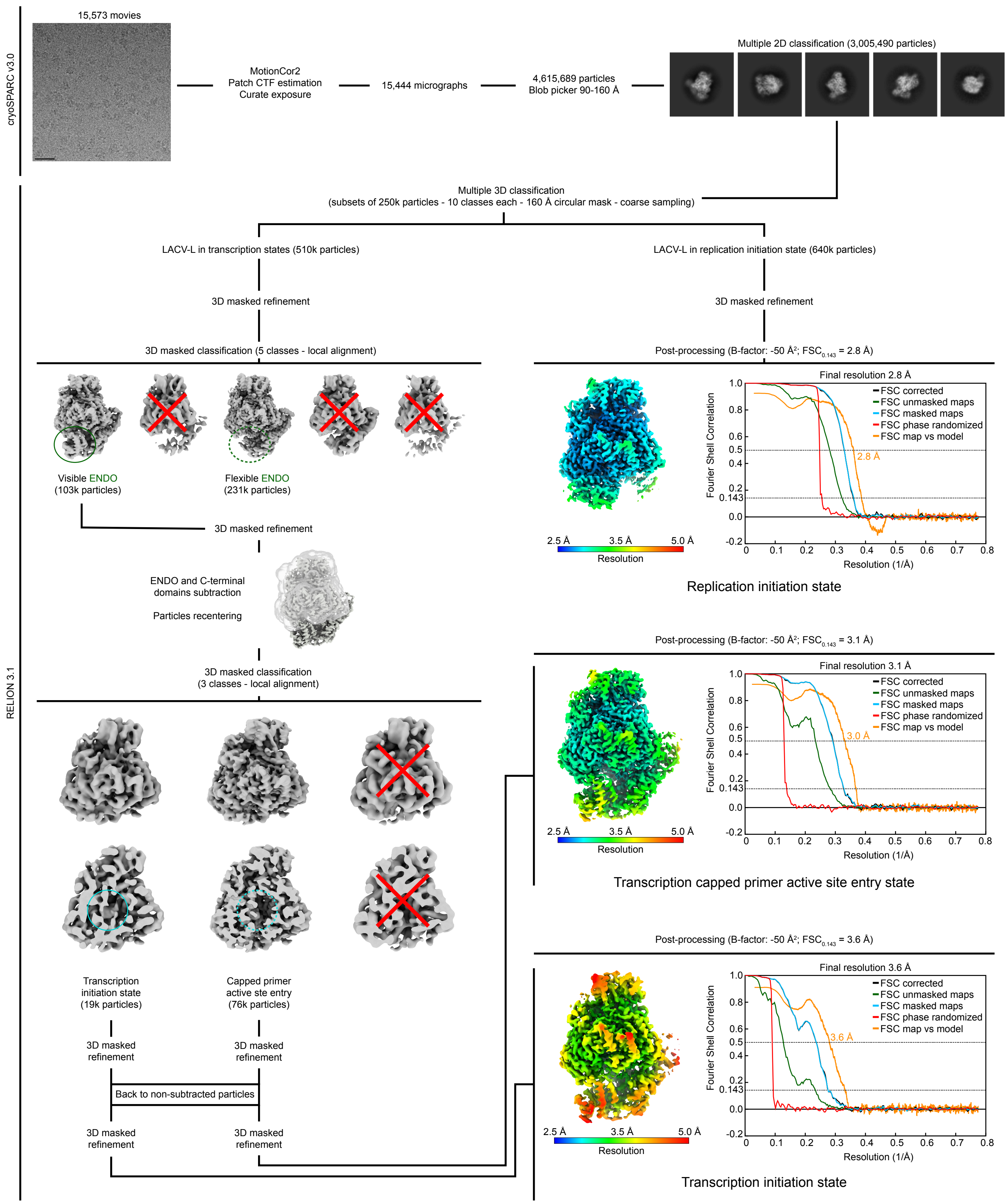

Supplementary Fig. 3. Image processing strategy to obtain the replication initiation state, the transcription capped primer active site entry state and the transcription initiation state
Schematics of the image processing strategy used with the data collected on a Titan Krios equipped a K3 direct electron detector. Representative micrograph, 2D class averages, 3D class averages are displayed. CTF stands for contrast transfer function, ENDO for endonuclease. Local resolution EM maps colored according to resolution are shown. Fourier shell correlation curves (FSC) are displayed. Scale bar = 200 Å.

SUPPLEMENTARY FIGURE 4

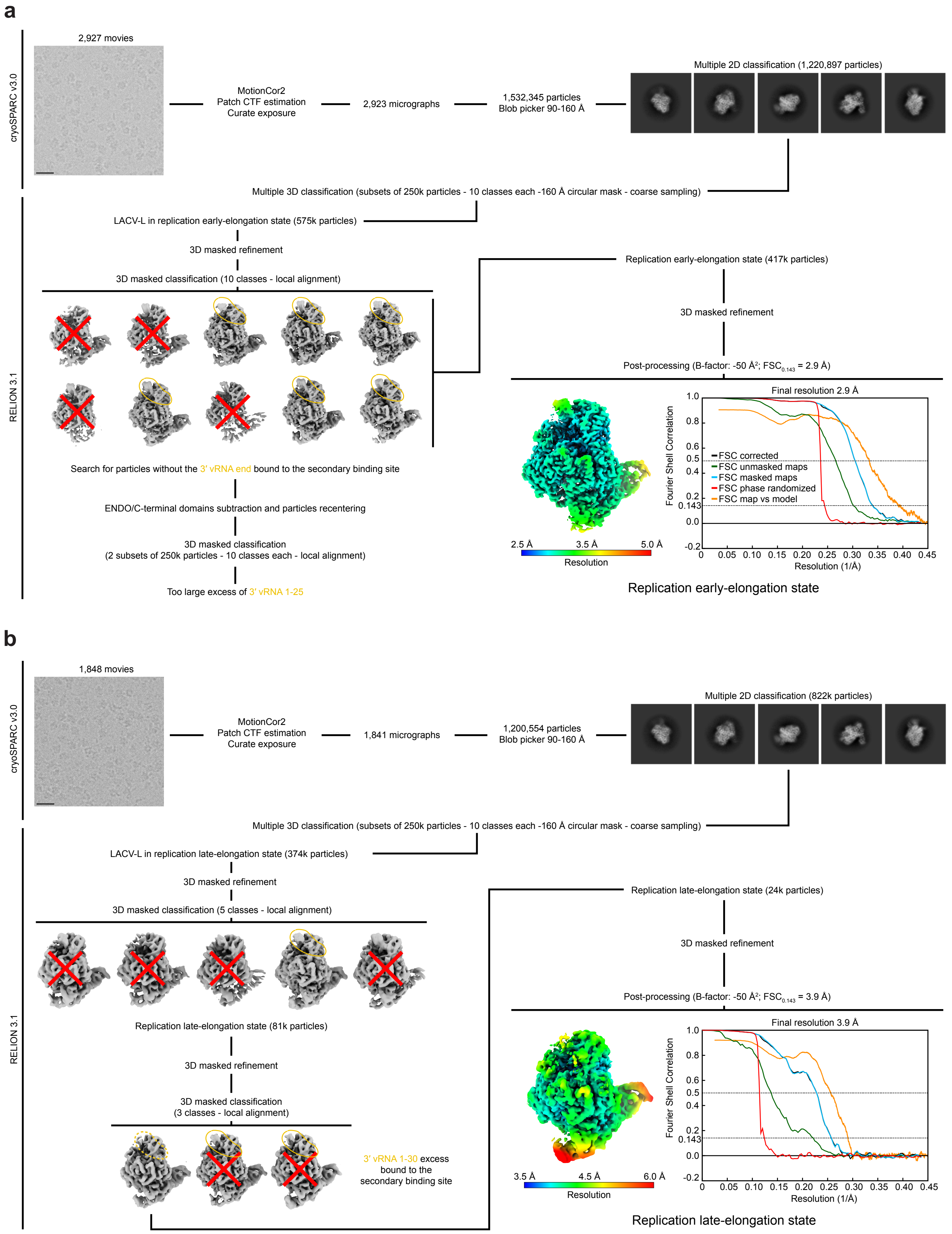

Supplementary Fig. 4. Image processing strategy to obtain the replication early-elongation state and the replication late-elongation state

a,b, Schematics of the image processing strategy used with the data collected on a Glacios cryo-TEM equipped a K2 direct electron detector to obtain the replication early-elongation state (a) and the replication late-elongation state (b). Representative micrographs, 2D class averages, 3D class averages are displayed. CTF stands for contrast transfer function, ENDO for endonuclease. Local resolution EM maps colored according to resolution are shown. Fourier shell correlation curves (FSC) are displayed. Scale bar = 200 Å.

SUPPLEMENTARY FIGURE 5

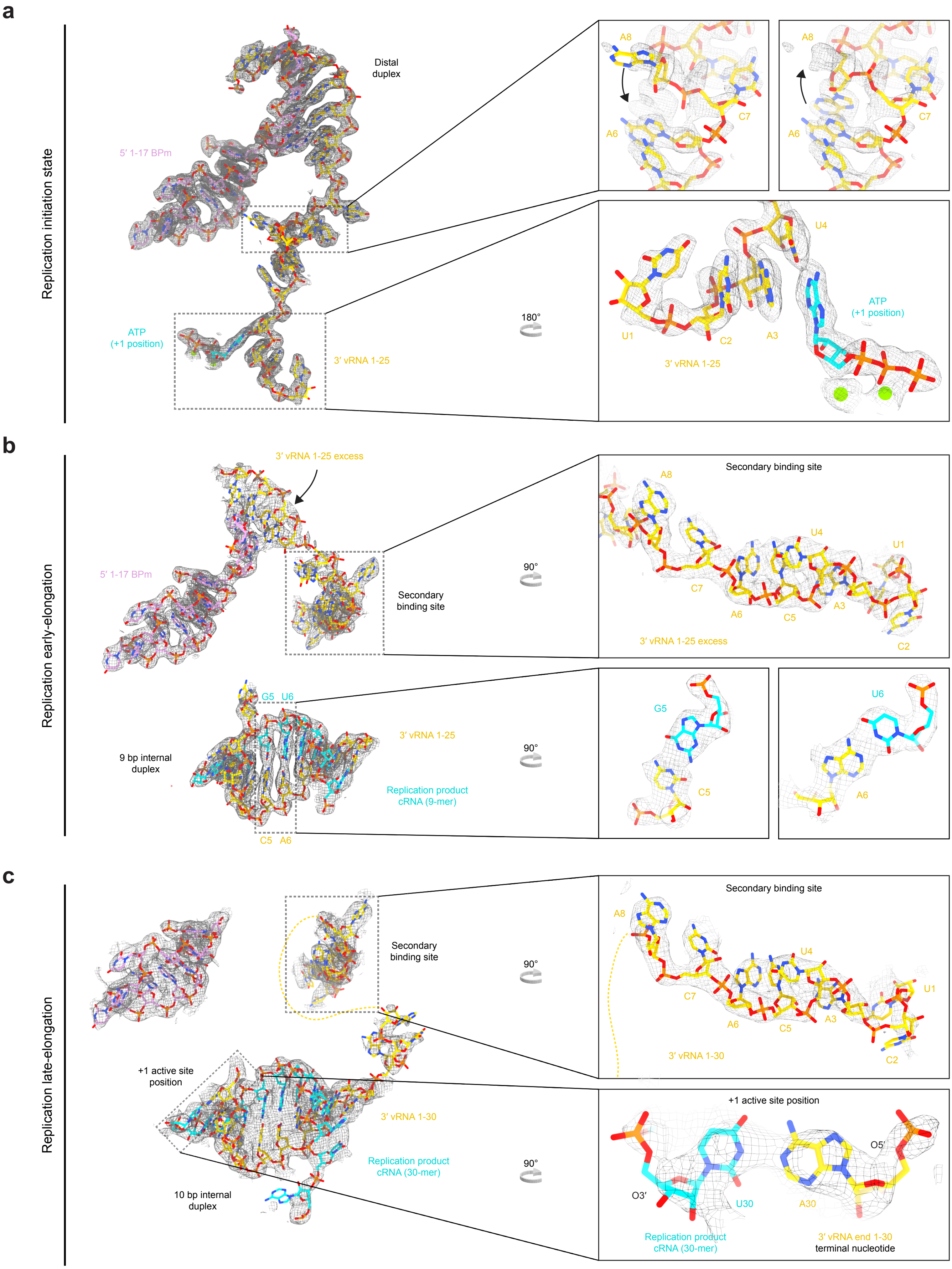

Supplementary Fig. 5. RNA densities and models in the different replication states.
a, (left) General view of the RNA density and model in the replication initiation state. (Right, top) Focused view of nucleotides A6, C7, A8 densities in the replication initiation state. Alternative conformations are visible in the density. A8 is built in one conformation in each zoom. An arrow indicates A8 movement. (Right, bottom) Focused view of the RNA density focusing on the ATP, the nucleotides and the magnesium ions present in the active site.
b, (left) General view of the RNA density and model at replication early-elongation. (Right, top) Focused view on the 3′-vRNA excess that binds in the 3′ secondary binding site. (Right, bottom) Focused view on some nucleotides of the RNA template/product duplex.
c, (left) General view of the RNA density and model at replication late-elongation. (Right, top) Zoom of the template 3′ end that binds to the 3′ secondary binding site. (Right, bottom) Zoom on the +1 active site position that identifies the template and product terminal nucleotides.

SUPPLEMENTARY FIGURE 6

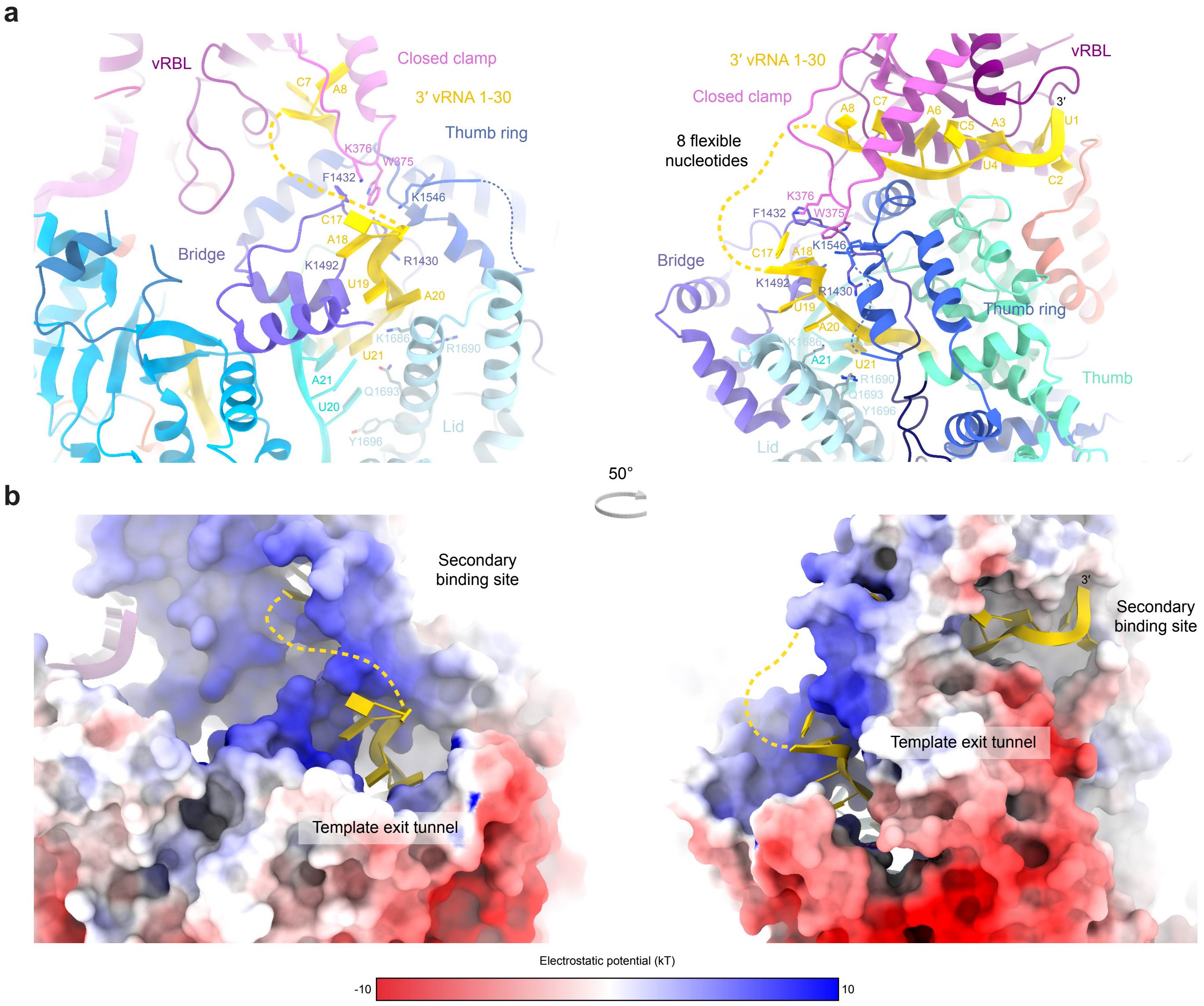

Supplementary Fig. 6. Path of the template RNA from the template exit channel to the 3′-vRNA secondary binding site

a, The 3′-vRNA is displayed in yellow. Interacting residues in the template exit channel are indicated. vRBL stands for vRNA binding lobe.

b, LACV-L$_{\text{Cltag_H34K}}$ surface colored according to its electrostatic potential showing that the path from the template exit channel towards the secondary binding site is positively charged.

SUPPLEMENTARY FIGURE 7

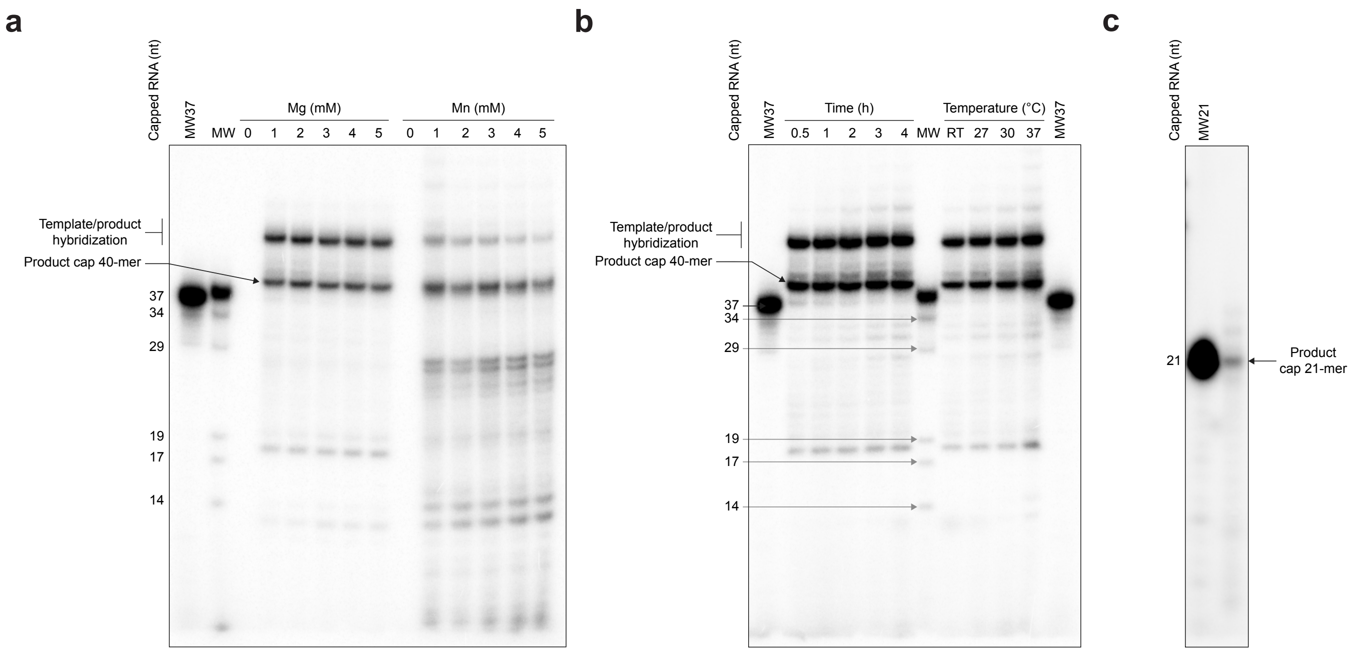

Supplementary Fig. 7. Transcription activity optimization of LACV-$L_{\text{CItag\_H34K}}$

a, Optimization of the divalent metal ion concentration for LACV-$L_{\text{CItag\_H34K}}$ transcription activity. Assessment of transcription product formation using concentration ranging from 0 to 5 mM of $MgCl_2$ or $MnCl_2$. The MW37 corresponds to a capped 37-mer RNA identical in sequence to the theoretical transcription product of LACV-$L_{\text{CItag\_H34K}}$ with the cap14AG and the 3′-vRNA1-25 in the absence of realignment. The MW corresponds to an RNAse T1 cleavage of the MW37. This experiment was repeated independently 2 times with similar results. Source data are provided as a Source Data file.

b, Time course and temperature effect on LACV-$L_{\text{CItag\_H34K}}$ transcription activity. Reactions were stopped after 0.5, 1, 2, 3 or 4h. Reactions were run at room temperature (RT), 27, 30 or 37°C. This experiment was repeated independently 2 times with similar results. Source data are provided as a Source Data file.

c, Transcription reaction of LACV-L when incubated with cap14AG, 3′-vRNA 1-25, 5′-1-17 BPm and ATP/GTP/UTP. The molecular weight marker (MW21) is a capped RNA with a sequence identical to the expected LACV-L un-realigned transcript product. Source data are provided as a Source Data file.

SUPPLEMENTARY FIGURE 8

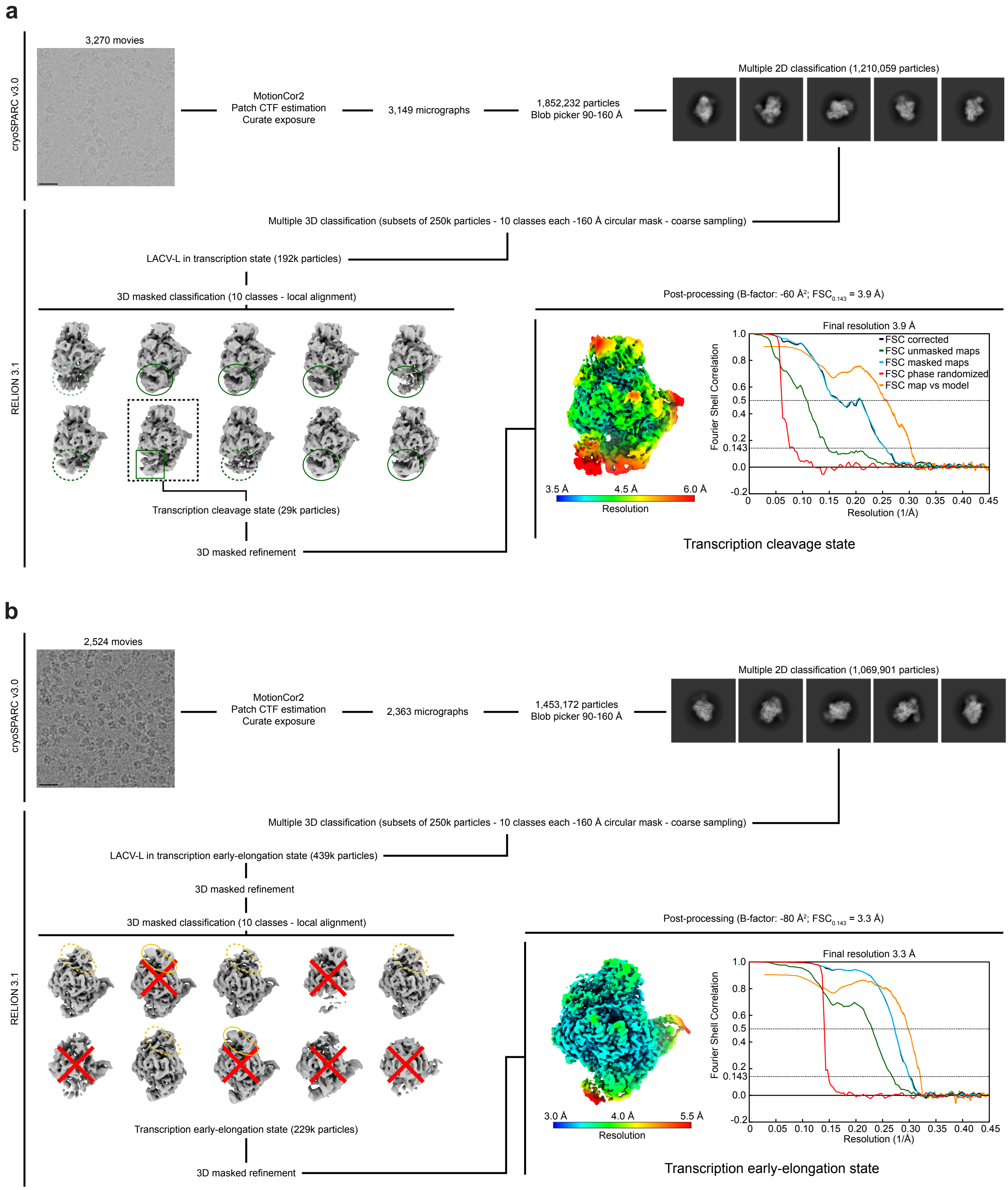

Supplementary Fig. 8. Image processing strategy to obtain the transcription capped primer cleavage state and the transcription early-elongation state
a,b, Schematics of the image processing strategy used with the data collected on a Glacios cryo-TEM equipped a K2 direct electron detector to obtain the transcription capped primer cleavage state (a) and the transcription early-elongation state (b). Representative micrographs, 2D class averages, 3D class averages are displayed. Local resolution EM maps colored according to resolution are shown. CTF stands for contrast transfer function. Fourier shell correlation (FSC) curves are displayed. Scale bar = 200 Å.

SUPPLEMENTARY FIGURE 9

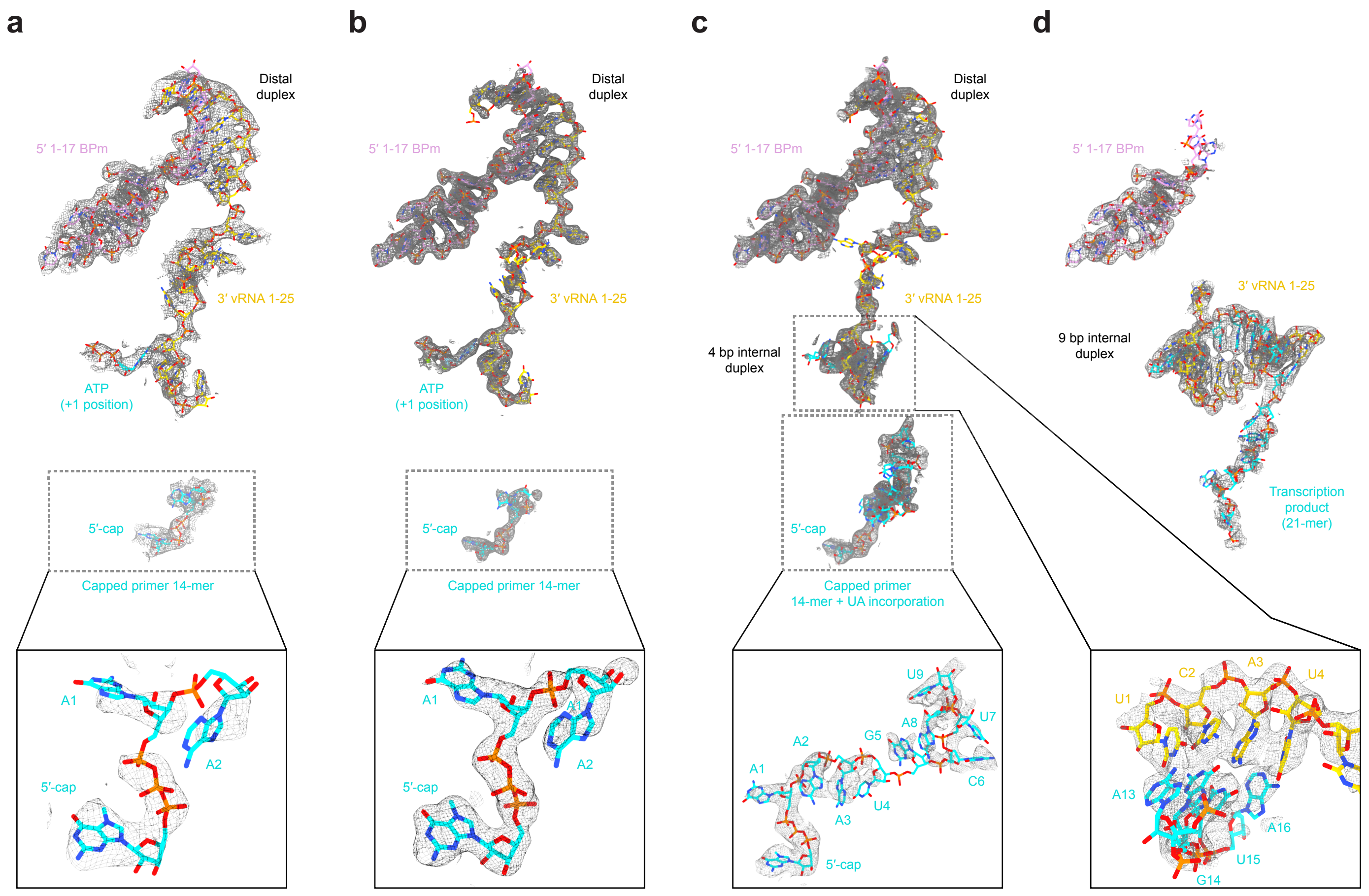

Supplementary Fig. 9. RNA densities and models in the different transcription states.
a, General view of the RNA density and model in the putative endonuclease cleavage conformation. Additional zoom on both capped RNA density and model is shown.
b, General view of the RNA density and model in the capped primer active site entry conformation. Additional zoom on both capped RNA density and model is shown.
c, General view of the RNA density and model in the transcription initiation conformation. Additional zoom on the capped RNA density, the 4 base-pair internal duplex density and their corresponding models are shown.
d, General view of the RNA density and model in the transcription early-elongation conformation.

SUPPLEMENTARY FIGURE 10

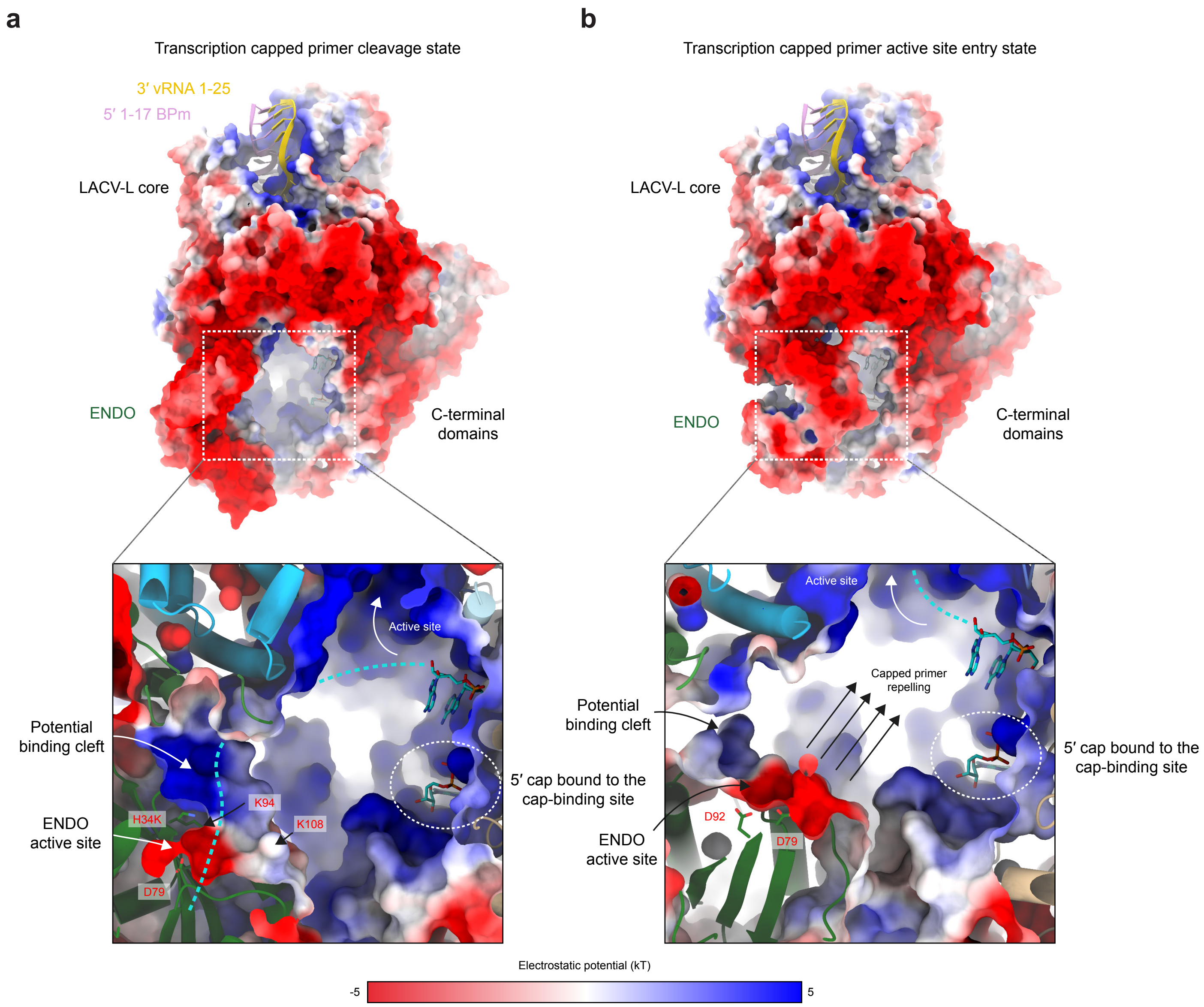

Supplementary Fig. 10. Electrostatic surface analysis of LACV-L_{Cltag_H34K} in the transcription capped primer cleavage state and the transcription capped primer active site entry state conformations
a, Electrostatic surface of the capped primer cleavage conformation. Top: global view, bottom: slabbed and zoomed view focusing on the charged environment close to the capped RNA. 5′-1-17BPm and 3′-vRNA-1-25 are displayed and respectively colored in pink and gold. RNAs tunnels are positively charged. The endonuclease (ENDO) is presenting a potential binding cleft for the capped primer (dotted line in cyan) surrounded by K94 and K108 leading to the ENDO active site.
b, Electrostatic surface of the capped primer active site entry conformation as in (a). RNAs tunnels are positively charged. The ENDO active site is repelling the capped primer (dotted line in cyan) into the RdRp active site.

SUPPLEMENTARY FIGURE 11

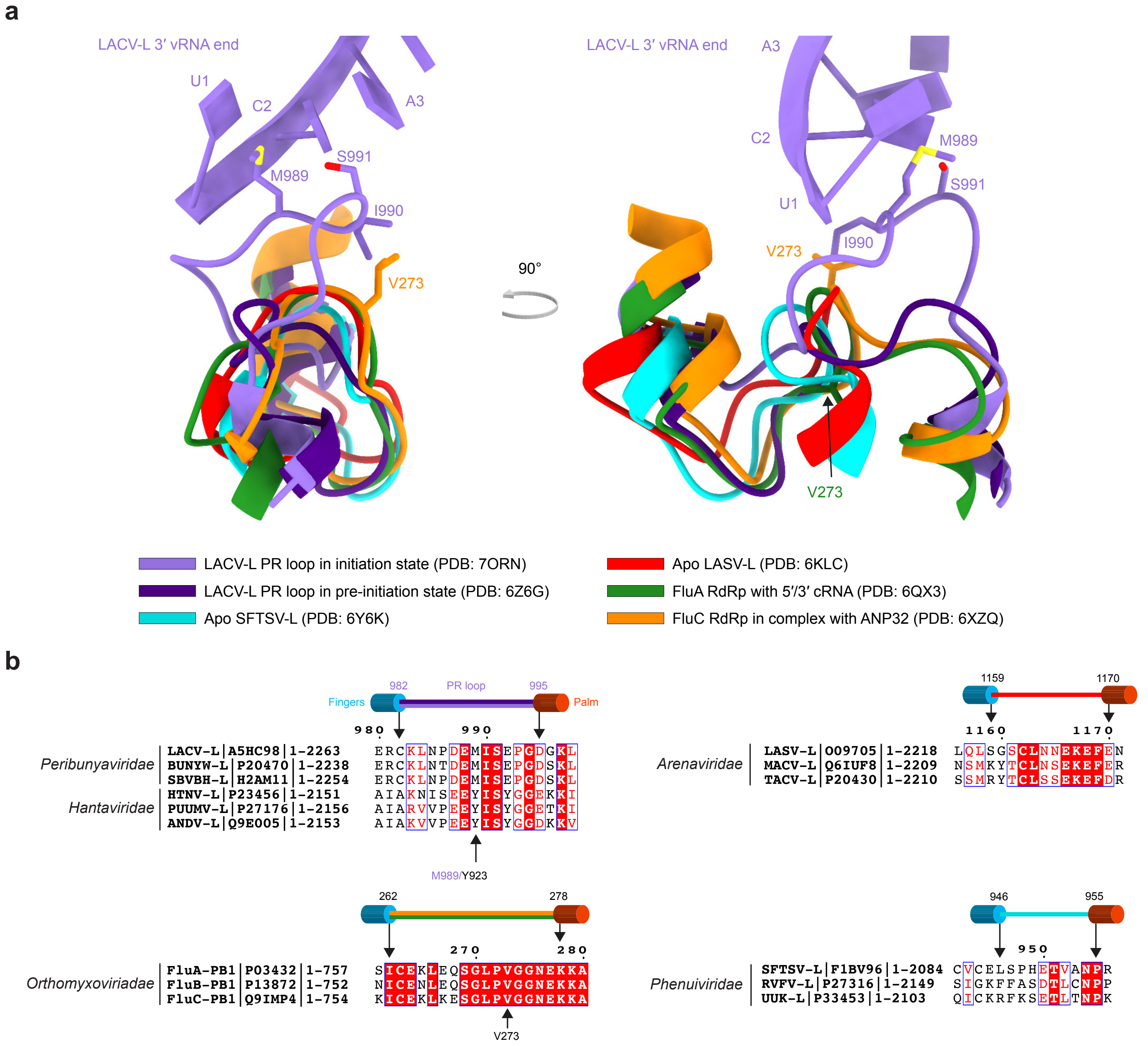

Supplementary Fig. 11. Putative Prime-and-realign loops in sNSV

a, Superposition of LACV-L prime-and-realign loop (PR loop) in initiation state (purple), pre-initiation state (dark blue, PDB: 6Z6G), apo SFTSV-L (946-955) (light blue, PDB: 6Y6K), apo LASV-L (1159-1170) (red, PDB: 6KLC), FluA RdRp (262-278) in complex with 5′- and 3′-cRNA (green, PDB: 6QX3) and FluC RdRp (262-278) in complex with ANP32 (orange, PDB: 6XZQ). Interacting residues of the LACV-L PR loop with the 3′-vRNA end (M989, I990, S991) and the Flu RdRp (V273) are displayed. SFTSV stands for Severe Fever with Thrombocytopenia Syndrome virus, LASV for Lassa virus, RdRp for RNA-dependent-RNA-polymerase.

b, Sequence alignment of the putative PR loop sequence in sNSV. Peribunyaviridae (LACV: La Crosse virus, BUNYW: Bunyamwera virus, SBVBH: Schmallenberg virus) and Hantaviridae (HTNV: Hantaan virus, PUUMV: Puumala virus, ANDV: Andes virus) families share some identity regarding LACV-L PR loop residues interacting with the 3′-vRNA template (E988, I990, S991). M989 in LACV-L is replaced by an aromatic residue (Y926) in Hantaviridae L protein that could play a similar role in template stabilization. Orthomyxoviruses (Influenza A, B, C) share a conserved PR loop on PB1 subunit with V273 playing an important role in prime-and-realign mechanism (Oymans and Te Velthuis, 2018). In Arenaviridae (LASV: Lassa virus, MACV: Machupo virus, TACV: Tacaribe virus), a conserved loop is also present between residues 1159 and 1170 contrary to Phenuiviridae (SFTSV: Sever Fever with Thrombocytopenia Syndrome virus, UUK: Uukuniemi virus) in which only T951 is conserved. For each displayed sequence, the virus name, the UniProt number and the viral polymerase length are indicated.

SUPPLEMENTARY FIGURE 12

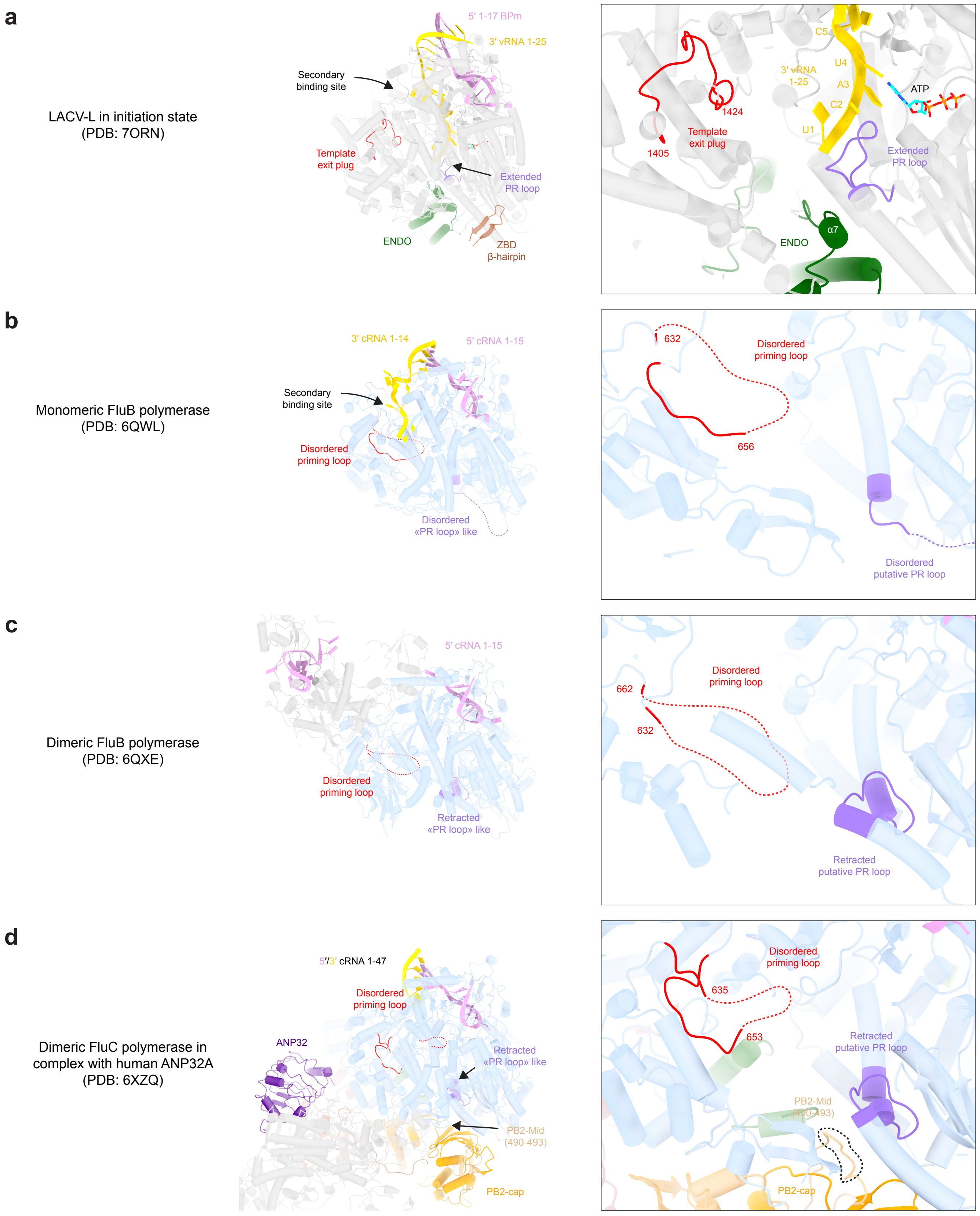

Supplementary Fig. 12. Prime-and-realign loops, LACV-L template exit plug and influenza polymerase priming loop
a-d, Cartoon representation of LACV-L in initiation state (a), monomeric influenza B polymerase bound to cRNA (PDB: 6QWL) (b), dimeric influenza B polymerase bound to cRNA (PDB: 6QXE) (c), dimeric influenza C polymerase in complex with human ANP32 (PDB: 6XZQ) (d). LACV-L core is transparent and colored in grey. Flu RdRp core is transparent and colored in blue. 3′-vRNA/cRNA end and 5′-vRNA/cRNA end present in each structure are respectively colored in gold and pink. The LACV-L template exit plug and the influenza priming loop are colored in red, if disordered represented as a dotted line. PR loops are colored in purple. Influenza PB2-cap is colored in orange, the PB2-mid in beige, both the PB2-627 and the LACV-L ZBD β-hairpin in brown. On the right panels, close-up view of the polymerase internal cavity.

SUPPLEMENTARY FIGURE 13

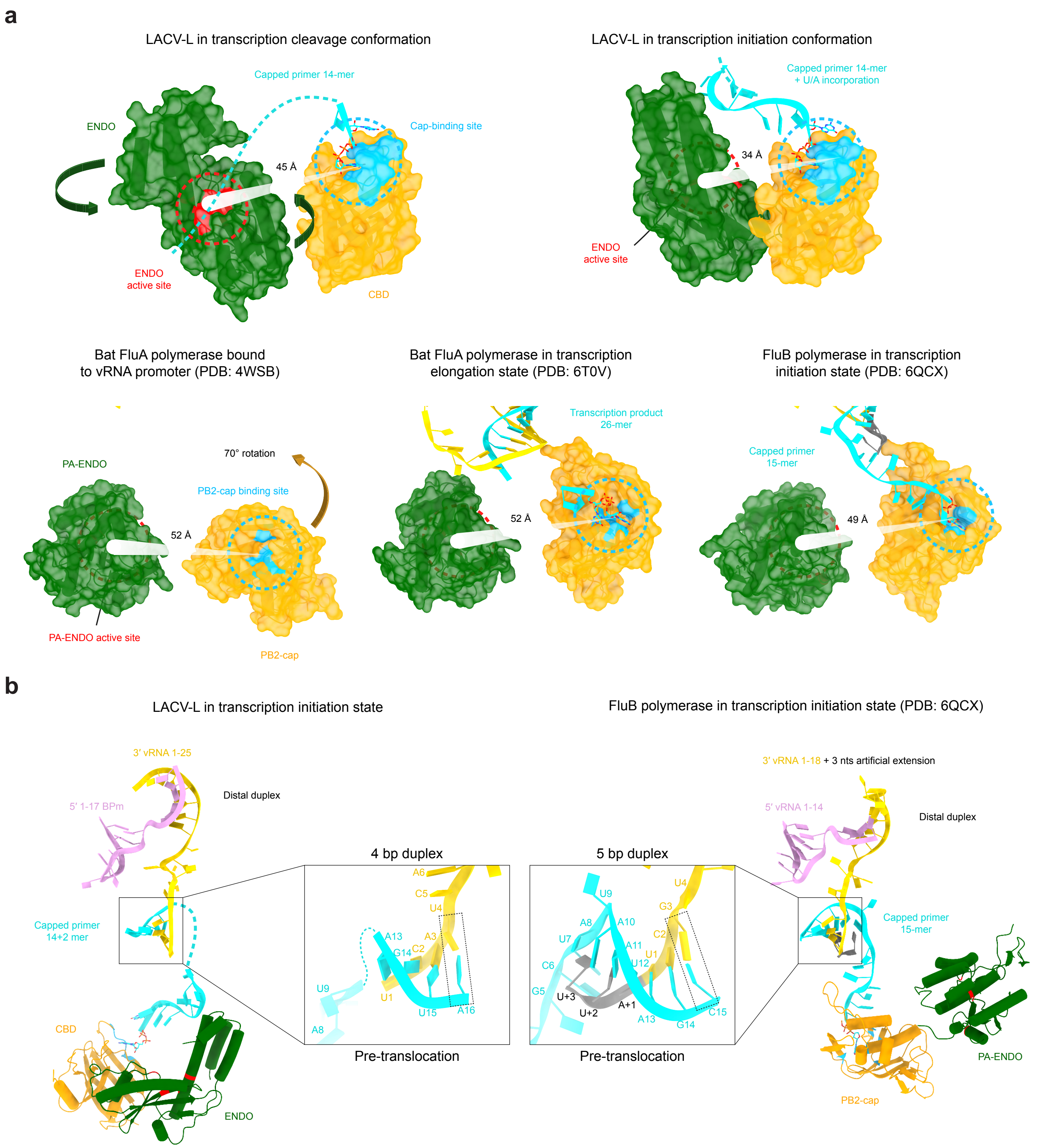

Supplementary Fig. 13. ENDO and CBD positions in LACV-L and influenza polymerase during transcription initiation

a, Surface representation of the endonuclease (ENDO) and the cap-binding domain (CBD) of LACV-L (top) and influenza polymerase (bottom). ENDOs are colored in green with active sites in red. CBDs are colored in yellow with cap-binding active sites in cyan. Distances between ENDO active sites and cap-binding active sites are indicated. The capped primer is shown in cyan and its possible path as a dotted line. Rotations between the cleavage conformation and the transcription initiation conformation are indicated with arrows.

b, Comparison of the paths taken by the 3′-vRNA and the capped RNA primers between LACV-L (left) and influenza B polymerase (right). Both CBD and ENDO position are displayed. A zoom is made on the nascent template-product duplexes. bp stands for base-pair.
