# Supplementary material for: Structural snapshots of La Crosse virus polymerase reveal the mechanisms underlying Peribunyaviridae replication and transcription
Source: Nat Commun. 2022 Feb 16;13:902. doi: 10.1038/s41467-022-28428-z (PMC8850483; doi:10.1038/s41467-022-28428-z)
Supplement: Supplementary file 3 — Description of Additional Supplementary Files [file 41467_2022_28428_MOESM3_ESM.pdf]

Description of Additional Supplementary Files

File Name: Supplementary Movie 1
Description: Replication: LACV-L from preinitiation to late-elongation

File Name: Supplementary Movie 2
Description: Transcription: LACV-L from preinitiation to early-elongation
