# Supplementary material for: Structural snapshots of La Crosse virus polymerase reveal the mechanisms underlying Peribunyaviridae replication and transcription
Source: Nat Commun. 2022 Feb 16;13:902. doi: 10.1038/s41467-022-28428-z (PMC8850483; doi:10.1038/s41467-022-28428-z)
Supplement: Supplementary file 6 — Reporting Summary [file 41467_2022_28428_MOESM6_ESM.pdf]

nature portfolio

Corresponding author(s): Hélène Malet, Stephen Cusack

Last updated by author(s): Jan 4, 2022

Reporting Summary

Nature Portfolio wishes to improve the reproducibility of the work that we publish. This form provides structure for consistency and transparency in reporting. For further information on Nature Portfolio policies, see our Editorial Policies and the Editorial Policy Checklist.

Statistics

For all statistical analyses, confirm that the following items are present in the figure legend, table legend, main text, or Methods section.

n/a	Confirmed	
☐	☒	The exact sample size (*n*) for each experimental group/condition, given as a discrete number and unit of measurement
☐	☒	A statement on whether measurements were taken from distinct samples or whether the same sample was measured repeatedly
☐	☒	The statistical test(s) used AND whether they are one- or two-sided<br>*Only common tests should be described solely by name; describe more complex techniques in the Methods section.*
☒	☐	A description of all covariates tested
☒	☐	A description of any assumptions or corrections, such as tests of normality and adjustment for multiple comparisons
☐	☒	A full description of the statistical parameters including central tendency (e.g. means) or other basic estimates (e.g. regression coefficient) AND variation (e.g. standard deviation) or associated estimates of uncertainty (e.g. confidence intervals)
☐	☒	For null hypothesis testing, the test statistic (e.g. *F*, *t*, *r*) with confidence intervals, effect sizes, degrees of freedom and *P* value noted<br>*Give P values as exact values whenever suitable.*
☒	☐	For Bayesian analysis, information on the choice of priors and Markov chain Monte Carlo settings
☒	☐	For hierarchical and complex designs, identification of the appropriate level for tests and full reporting of outcomes
☒	☐	Estimates of effect sizes (e.g. Cohen's *d*, Pearson's *r*), indicating how they were calculated

Our web collection on statistics for biologists contains articles on many of the points above.

Software and code

Policy information about availability of computer code

Data collection	SerialEM 3.8.0b, EPU2.6
Data analysis	MotionCor2 1.4.0, CryoSPARC v3.2.0, Relion3.1, COOT, Phenix 1.19.2-4158, Chimera 1.15, ChimeraX 1.3, PDB2PQR 2.1, APBS 1.4.0, muscle, EsPript, NR-grep 1.1.2, GNU grep 2.20, Cutadapt 3.4, GNU AWK 4.02, ImageJ 1.53K, R 4.1.1.

For manuscripts utilizing custom algorithms or software that are central to the research but not yet described in published literature, software must be made available to editors and reviewers. We strongly encourage code deposition in a community repository (e.g. GitHub). See the Nature Portfolio guidelines for submitting code & software for further information.

Data

Policy information about availability of data

All manuscripts must include a data availability statement. This statement should provide the following information, where applicable:

- Accession codes, unique identifiers, or web links for publicly available datasets
- A description of any restrictions on data availability
- For clinical datasets or third party data, please ensure that the statement adheres to our policy

The coordinates and EM maps generated in this study have been deposited in the Protein Data Bank and the Electron Microscopy Data Bank under accession codes:
LACV-L replication initiation state PDB 7ORN EMDB EMD-13043
LACV-L replication early-elongation state PDB 7ORO EMDB EMD-13044
LACV-L replication late-elongation state PDB 7ORI EMDB EMD-13038
LACV-L transcription capped primer cleavage state PDB 7ORJ EMDB EMD-13039
LACV-L transcription capped primer active site entry state PDB 7ORK EMDB EMD-13040

LACV-L transcription initiation state PDB 7ORL EMDB EMD-13041
LACV-L transcription early-elongation state PDB 7ORM EMDB EMD-13042
The NGS raw reads generated in this study have been deposited in the European nucleotide archive under accession code ERP132950.
Source data are provided with this paper.
The coordinates and EM maps used in this study are available in the Protein Data Bank and the Electron Microscopy Data Bank under accession codes:
LACV-L pre-initiation state PDB 6Z6G EMDB EMD-11093
LACV-L elongation-mimicking state PDB 6Z8K EMD-11118

Field-specific reporting

Please select the one below that is the best fit for your research. If you are not sure, read the appropriate sections before making your selection.

- [x] Life sciences
- [ ] Behavioural & social sciences
- [ ] Ecological, evolutionary & environmental sciences

For a reference copy of the document with all sections, see nature.com/documents/nr-reporting-summary-flat.pdf

Life sciences study design

All studies must disclose on these points even when the disclosure is negative.

Sample size

The number of particles extracted for each of the EM reconstruction has been stated in the methods section. Each dataset was collected for two days on Krios (maximum beam time allocated) or three days on Glacios (manimum beam time allocated) with optimized pre-screened concentrated grids in order to provide the maximum number of particle possible at the time of the data collection.
For Fig. 2e the gels have been repeated three times with distinct samples. This number is chosen to ensure that the gels used for quantification are run within the same week, a time period during which we can consider the P32 source radioactivity as being constant.

Data exclusions

No data were excluded from the analyses

Replication

Protein expression, purification were performed 10 times and gave the same result.
In vitro replication and transcription assays were done between two and three times and gave the same results
Data collection of equilavent samples were performed two times and provided the same conclusions. The data at the highest resolution is provided.
For Fig. 2e the gels have been repeated three times with distinct samples and were reproducible.

Randomization

Extracted particles were randomly assigned to two separate groups to calculate half-maps and gold-standard FSC.

Blinding

Blinding was not applicable since predetermined samples and conditions were used throughout the study.

Reporting for specific materials, systems and methods

We require information from authors about some types of materials, experimental systems and methods used in many studies. Here, indicate whether each material, system or method listed is relevant to your study. If you are not sure if a list item applies to your research, read the appropriate section before selecting a response.

Materials & experimental systems

n/a	Involved in the study	
x		Antibodies
	x	Eukaryotic cell lines
x		Palaeontology and archaeology
x		Animals and other organisms
x		Human research participants
x		Clinical data
x		Dual use research of concern

Methods

n/a	Involved in the study	
x		ChIP-seq
x		Flow cytometry
x		MRI-based neuroimaging

Eukaryotic cell lines

Policy information about cell lines

Cell line source(s)	HEK-293 and Hi5 cells were bought from Thermofisher Cat.# 85120602 (HEK-293) and Cat.# B85502 (Hi5)
Authentication	None of the cells were authenticated
Mycoplasma contamination	Tested negative for mycoplasma
Commonly misidentified lines (See ICLAC register)	*Name any commonly misidentified cell lines used in the study and provide a rationale for their use.*
